# The International Virus Bioinformatics Meeting 2022

**DOI:** 10.3390/v14050973

**Published:** 2022-05-05

**Authors:** Franziska Hufsky, Denis Beslic, Dimitri Boeckaerts, Sebastian Duchene, Enrique González-Tortuero, Andreas J. Gruber, Jiarong Guo, Daan Jansen, John Juma, Kunaphas Kongkitimanon, Antoni Luque, Muriel Ritsch, Gabriel Lencioni Lovate, Luca Nishimura, Célia Pas, Esteban Domingo, Emma Hodcroft, Philippe Lemey, Matthew B. Sullivan, Friedemann Weber, Fernando González-Candelas, Sarah Krautwurst, Alba Pérez-Cataluña, Walter Randazzo, Gloria Sánchez, Manja Marz

**Affiliations:** 1 European Virus Bioinformatics Center, 07743 Jena, Germany; e.gonzaleztortuero@salford.ac.uk (E.G.-T.); andreas.j.gruber@uni-konstanz.de (A.J.G.); guo.1773@osu.edu (J.G.); daan.jansen@kuleuven.be (D.J.); kongkitimanonk@rki.de (K.K.); aluque@sdsu.edu (A.L.); anne.muriel.christin.ritsch@uni-jena.de (M.R.); gabriel.lencioni.lovate@uni-jena.de (G.L.L.); rnishimura@nig.ac.jp (L.N.); edomingo@cbm.csic.es (E.D.); emma.hodcroft@ispm.unibe.ch (E.H.); philippe.lemey@kuleuven.be (P.L.); sullivan.948@osu.edu (M.B.S.); friedemann.weber@vetmed.uni-giessen.de (F.W.); fernando.gonzalez@uv.es (F.G.-C.); alba.perez@iata.csic.es (A.P.-C.); wrandazzo@iata.csic.es (W.R.); gloriasanchez@iata.csic.es (G.S.); 2 RNA Bioinformatics and High-Throughput Analysis, Friedrich Schiller University Jena, 07743 Jena, Germany; sarah.krautwurst@uni-jena.de; 3 Methodology and Research Infrastructure, MF1 Bioinformatics, Robert Koch Institute, 13353 Berlin, Germany; beslicd@rki.de; 4 Laboratory of Applied Biotechnology, Department of Biotechnology, Ghent University, 9000 Ghent, Belgium; dimitri.boeckaerts@ugent.be (D.B.); celia.pas@ugent.be (C.P.); 5 KERMIT, Department of Data Analysis and Mathematical Modelling, Ghent University, 9000 Ghent, Belgium; 6 Peter Doherty Institute for Infection and Immunity, University of Melbourne, Melbourne 3000, Australia; sebastian.duchene@unimelb.edu.au; 7 School of Science, Engineering and Environment (SEE), University of Salford, Salford M5 4WT, UK; 8 Department of Biology, University of Konstanz, 78464 Konstanz, Germany; 9 Departments of Microbiology, and Civil, Environmental, and Geodetic Engineering, Ohio State University, Columbus, OH 43210, USA; 10 Department of Microbiology, Immunology and Transplantation, Rega Institute, Laboratory of Viral Metagenomics, KU Leuven, 3000 Leuven, Belgium; 11 International Livestock Research Institute (ILRI), Nairobi 00100, Kenya; j.juma@cgiar.org; 12 South African National Bioinformatics Institute, South African MRC Bioinformatics Unit, Cape Town 7530, South Africa; 13 Viral Information Institute, San Diego State University, San Diego, CA 92116, USA; 14 Computational Science Research Center, San Diego State University, San Diego, CA 92116, USA; 15 Department of Mathematics and Statistics, San Diego State University, San Diego, CA 92116, USA; 16 JRG Analytical MicroBioinformatics, Friedrich Schiller University Jena, 07743 Jena, Germany; 17 Department of Genetics, School of Life Science, The Graduate University for Advanced Studies (SOKENDAI), Mishima 411-8540, Japan; 18 Human Genetics Laboratory, National Institute of Genetics, Mishima 411-8540, Japan; 19 Centro de Biología Molecular “Severo Ochoa” (CSIC-UAM), 28049 Madrid, Spain; 20 Centro de Investigación Biomédica en Red de Enfermedades Hepáticas y Digestivas (CIBERehd) del Instituto de Salud Carlos III, 28029 Madrid, Spain; 21 Institute of Social and Preventive Medicine, University of Bern, 3012 Bern, Switzerland; 22 Swiss Institute of Bioinformatics, 1015 Lausanne, Switzerland; 23 Department of Microbiology, Immunology and Transplantation, Rega Institute, KU Leuven, 3000 Leuven, Belgium; 24 Institute for Virology, Veterinary Medicine, Justus-Liebig University, 35390 Gießen, Germany; 25 Joint Research Unit “Infection and Public Health” FISABIO, University of Valencia, 46010 Valencia, Spain; 26 Institute for Integrative Systems Biology (I2SysBio), CSIC, University of Valencia, 46010 Valencia, Spain; 27 VISAFELab, Department of Preservation and Food Safety Technologies, Institute of Agrochemistry and Food Technology, IATA-CSIC, 46980 Valencia, Spain

**Keywords:** bioinformatics, tools, SARS-CoV-2, viral emergence and surveillance, virus–host interactions, viral sequence analysis, virus identification and annotations, phages, viral diversity

## Abstract

The International Virus Bioinformatics Meeting 2022 took place online, on 23–25 March 2022, and has attracted about 380 participants from all over the world. The goal of the meeting was to provide a meaningful and interactive scientific environment to promote discussion and collaboration and to inspire and suggest new research directions and questions. The participants created a highly interactive scientific environment even without physical face-to-face interactions. This meeting is a focal point to gain an insight into the state-of-the-art of the virus bioinformatics research landscape and to interact with researchers in the forefront as well as aspiring young scientists. The meeting featured eight invited and 18 contributed talks in eight sessions on three days, as well as 52 posters, which were presented during three virtual poster sessions. The main topics were: SARS-CoV-2, viral emergence and surveillance, virus–host interactions, viral sequence analysis, virus identification and annotation, phages, and viral diversity. This report summarizes the main research findings and highlights presented at the meeting.

## 1. Introduction

The International Virus Bioinformatics Meeting (ViBioM) was the fifth edition of the virus bioinformatics meeting organized by the European Virus Bioinformatics Center (EVBC). The EVBC was founded in 2017 to bring together experts in virology and virus bioinformatics in Europe [1,2]. The EVBC is constantly growing, having currently 245 members (∼30% increase since the last meeting in 2020 [3]) from 140 research institutes distributed over 36 countries worldwide.

ViBioM 2022 (please note that we had to change the abbreviation of the event from IVBM to ViBioM to not be confused with the International Vascular Biology Meeting) was planned to take place in Valencia, Spain in March 2022. As the number of Omicron cases in Europe were rapidly increasing in January 2022, we decided to again switch to an online format to avoid creating a transimission hotspot for SARS-CoV-2 and to make the conference planning less complicated (in terms of safety regulations and travel restrictions).

Virtual meetings have several advantages and disadvantages. The flexibility of listening to selected talks and not being compelled to travel (in particular, long distance) makes online meetings accessible to a broader range of scientists. Therefore, we had an incredibly high amount of registered participants. Even during the ongoing conference there were registrations up to the last day. There was a lot of fluctuation among the 380 registered participants and not everyone attended all talks. However, we had a solid base between 100–150 participants during each of the talks. From all registered participants, ∼19% are EVBC members; thus, ViBioM is attracting scientists far beyond the EVBC community. As in 2020, the participants made it possible to create a highly interactive scientific environment even without physical face-to-face interactions. Breakout rooms of the speakers for continued discussion during the coffee breaks were extensively used.

Another benefit of the virtual format was the flexibility we had when putting the program together. Due to the high amount of submissions on SARS-CoV-2-related research, we decided to add an additional conference day solely focusing on SARS-CoV-2. The extended meeting took place 23–25 March 2022. In total, the meeting featured eight invited and 18 contributed talks in eight sessions on three days, as well as 52 posters, which were presented during three virtual poster sessions.

## 2. Scientific Program

A number of high-quality presentations were given by leading experts and junior scientists on several different topics in virus bioinformatics. From over 50 submissions (a ∼25% increase compared to 2020 [3]), we selected 18 talks (acceptance rate: ∼35%). Due to the high amount of submissions on SARS-CoV-2-related research, we decided to add an additional conference day. On the first day, we were solely focusing on SARS-CoV-2-related research (see Section 2.1). On day two, we had three sessions on viral emergence and surveillance (see Section 2.2), virus-host interactions (see Section 2.3), and viral sequence analysis (see Section 2.4). On day three, we had three sessions on virus identification and annotation (see Section 2.5), phages (see Section 2.6), and viral diversity (see Section 2.7). Spyros Lytras (Exploring the dinucleotide composition of the Flaviviridae with DinuQ) was selected for Best ECR Talk Award.

During three virtual poster sessions, 52 posters were presented. In order to imitate onsite poster sessions, we set up a breakout room for each poster. Participants were free to visit and switch between the breakout rooms at all times during the poster session. The posters were made available beforehand. Three presenters were selected for the Best Poster Award: Gabriel Lencioni Lovate (Reproducible RNA–RNA interaction probing for RNA proximity ligation data with RNAswarm; see Section 2.4.3), Célia Pas (A blueprint of tail fiber modularity and its relationship with host specificity for STEC serovars; see Section 2.6.3), and Luca Nishimura (Virome analyses of the ancient individuals who lived in the Japanese archipelago 3000 years ago; see Section 2.7.3).

### 2.1. Satellite Meeting on SARS-CoV-2

Due to the high number of submissions on SARS-CoV-2, we decided to add a conference day with two additional sessions focusing on this virus. The sessions were hosted by EVBC member Martin Hölzer (Robert Koch Institute, Berlin, Germany) and Fernando González-Candelas (University of Valencia, Valencia, Spain), one of the ViBioM 2022 organizers. Two keynote speakers were invited on this topic: Francois Balloux (University College London, UK) opened the conference with a talk about the changing landscape of SARS-CoV-2 genetic diversity; and Philippe Lemey (KU Leuven, Belgium) opened the afternoon session and spoke about SARS-CoV-2 genomic epidemiology (see Section 2.1.1). From the submitted abstracts, we selected talks by Alice Wittig (Hasso Plattner Institute/Robert Koch Institute, Germany) about efficient and rapid genome profiling of SARS-CoV-2 sequences; Sebastian Duchene (University of Melbourne, Australia) about the emergence of SARS-CoV-2 variants of concern (see Section 2.1.2); Kunaphas Kongkitimanon (Robert Koch Institute, Germany) about an early warning system to detect concerning new SARS-CoV-2 variants from sequencing data (see Section 2.1.3); Francisco Ortuño (Fundación Progreso y Salud, Spain) about a new tool for the whole-genome imputation of SARS-CoV-2; Fabian Amman (CeMM—Forschungszentrum für Molekulare Medizin, Austria) about a national-scale surveillance of emerging SARS-CoV-2 variants in wastewater; and Denis Beslic (Robert Koch Institute, Germany) about the power of SARS-CoV-2 genotyping and SNP-based clustering for contextual outbreak assessment (see Section 2.1.4).

#### 2.1.1. SARS-CoV-2 Genomic Epidemiology: Bayesian Phylodynamic Reconstruction, Vaccine Design, and Characterization of Antigenic Evolution (by Philippe Lemey)

As the COVID-19 pandemic unfolded, viral genomic data was produced at an unprecedented scale, allowing us to track the SARS-CoV-2 evolutionary and epidemiological dynamics and providing important insights for intervention strategies. Here, I will highlight a number of developments in a Bayesian statistical framework in support of SARS-CoV-2 phylodynamic reconstructions, including the integration of individual travel history and mobility data [4] and its application to track the early introduction and spread of the virus [5]. The data integration concept has also been applied to the fullest when assessing the contribution of persistence and introductions in the second COVID-19 wave in Europe [6]. Finally, I will illustrate how genomic epidemiology has contributed to vaccine development at the Rega Institute. This involves the development of a COVID-19 vaccine using the yellow fever vaccine YF17D as a vector [7] that was updated based on evolutionary analyses of SARS-CoV-2 variants of concerns (VoC) [8]. While the original vaccine was able to bring down infectious virus loads to undetectable levels for both the prototype virus from early 2020 as well as for VoC alpha in a hamster model, the immunity elicited against VoC beta was insufficient to provide optimal protection. An updated vaccine using the gamma spike protein offers efficient protection against lower respiratory tract infection and COVID-19-like pathology for VOC alpha, beta, gamma and delta [9]. We demonstrate how antigenic cartography based on seroneutralization assays is able to map the antigenic divergence for these VoCs. Moreover, for the recent omicron VoC, the updated vaccine resulted in a considerably higher degree of seroneutralization. Antigenic mapping indicates a far more pronounced antigenic divergence for this VoC.

#### 2.1.2. The Emergence of SARS-CoV-2 Variants of Concern Is Driven by Acceleration of the Substitution Rate (by Sebastian Duchene)

The ongoing SARS-CoV-2 pandemic has observed an unprecedented amount of rapidly generated genome data. These data have revealed the emergence of lineages with mutations associated to transmissibility and antigenicity, known as variants of concern (VOCs). A striking aspect of VOCs is that many of them involve an unusually large number of defining mutations. Current phylogenetic estimates of the substitution rate of SARS-CoV-2 suggest that its genome accrues around 2 mutations per month. However, VOCs can have 15 or more defining mutations, and it is hypothesised that they emerged over the course of a few months, implying that they must have evolved faster for a period of time.

In this talk I will present detailed molecular clock analyses of genome sequence data from the GISAID database to assess whether the emergence of VOCs can be attributed to changes in the substitution rate of the virus.

Our results indicate that the emergence of VOCs is driven by an episodic increase in the substitution rate of around 4-fold of the background phylogenetic rate estimate that may have lasted several weeks or months. This outcome stands in contrast with the notion that the virus has overall increased its mutation rate. In sum, this study underscores the importance of monitoring the molecular evolution of the virus as a means of understanding the circumstances under which VOCs may emerge.

#### 2.1.3. VOCAL: An Early Warning System to Detect Concerning New SARS-CoV-2 Variants from Sequencing Data (by Kunaphas Kongkitimanon)

*Kunaphas Kongkitimanon, Martin Hölzer, and Hugues Richard contributed to this work.*

The evolution of the SARS-CoV-2 virus has demonstrated the emergence of waves of variants that reveal more worrying phenotypes, e.g., resulting in higher antibody escape or transmissibility [10]. Many new variants are observed and annotated as variants of interest or concern (VOI/C), e.g., by the WHO. However, delays in sequencing and case reporting and limited sampling capacity can make their identification lag weeks behind their emergence in the population. Hence, automated systems that could score concerning samples based on their sequence information and independently of their lineage assignment are highly needed. Furthermore, based on the extent of the convergent evolution observed in SARS-CoV-2, automated systems could generalize from previous examples to rank and identify potential concerning samples based on their amino acid (AA) profile.

**Figure 1:**
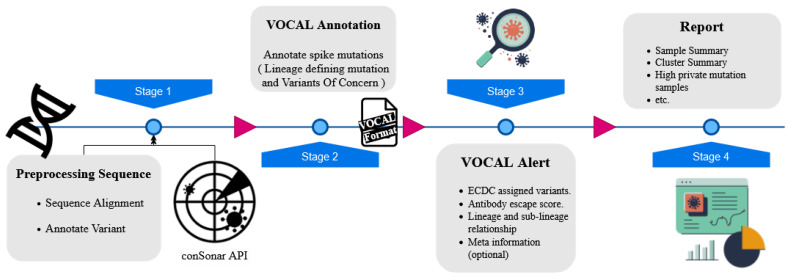
Main steps of the VOCAL pipeline. To generate mutation profiles for the spike gene for each input sequence, VOCAL can preprocess raw genome sequences or can directly receive this information from the covSonar.

Here, we present VOCAL, the Variant Of Concern ALert system, that can detect new emerging variants of SARS-CoV-2 and assign each variant to an alert level. VOCAL starts from complete genome sequences and categorizes the AA changes appearing in the spike protein depending on the type of non-synonymous mutations present and their overlap with known antibody binding sites, epitope regions, and correlation with antibody escape scores from deep mutational screens [11]. In addition, the tool provides an option to skip the alignment step and directly work on already pre-computed mutation profiles derived from covSonar developed at the RKI (https://github.com/rki-mf1/covsonar (accessed on 1 May 2022)). Based on the mutation profiles, VOCAL then detects the potentially concerning samples and ranks them according to three tiers of alert level: high, moderate, and low impact. Finally, VOCAL combines and summarizes all results in an HTML report to help users to investigate the raised alerts quickly (see Figure 1).

We retrospectively assessed the prediction power of VOCAL by considering all German sequences (https://zenodo.org/record/6409154 (accessed on 1 May 2022)) during two scenarios of emerging VOCs in 2021: the Delta variant (April) and the recent Omicron (December). Our testing set demonstrated that all of the VOC samples were correctly detected as a high concern (Delta: 30/30 (100%); Omicron: 3372/3446 (97%)). For Delta, we detected an additional set of 21 samples, which were mainly assigned to lineages B.1.617.1 and B.1.617.3 and have also been reported as concerning during that time. In conclusion, VOCAL is a specialized tool for the early detection of potentially concerning variants from enormous collections of SARS-CoV-2 genomes. The tool is freely available as stand-alone annotation and visualization or as a comprehensive workflow for molecular surveillance (https://github.com/rki-mf1/vocal (accessed on 3 May 2022)).

#### 2.1.4. The Power of SARS-CoV-2 Genotyping and SNP-Based Clustering for Contextual Outbreak Assessment (by Denis Beslic)

*Denis Beslic, Matthew Huska, Martin Hölzer, Sandra Kaiser, Hugues Richard, and Stephan Fuchs contributed to this work.*

The COVID-19 pandemic has triggered an unprecedented increase in viral genome sequencing for molecular surveillance. Between January 2021 and April 2022, over 800,000 SARS-CoV-2 genomes have been sequenced in Germany and over 10 million genomes have been uploaded to the international GISAID EpiCov database [12]. These datasets are ideally suited for potential outbreak identification but also to enrich and better understand local outbreak events with the additional associated sequences. Using the genetic distance of different samples to analyze their epidemiological relatedness has become an essential method for monitoring transmissions of various pathogens [13,14]. However, existing approaches are computationally costly and impractical given the current amount of data [15].

**Figure 2:**
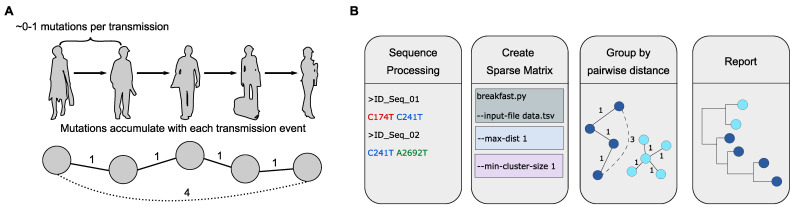
The power of SARS-CoV-2 genotyping and SNP-based clustering for contextual outbreak assessment. (**A**) We expect the viral genomes to accumulate mutations, as they are transmitted from one individual to the next. An efficient method to identify chains of genetically similar sequences would therefore be useful to identify putative outbreaks. (**B**) Diagram illustrating the steps of the clustering algorithm using a maximum SNP distance of 1. The mutation profiles are obtained by a reference-based alignment with Nextclade [17] or covSonar (https://github.com/rki-mf1/covsonar (accessed on 3 May 2022)). The pairwise distances between different sequences are derived from the constructed distance matrix of genomic profiles. Two sequences are part of the same transmission cluster if the pairwise distance between them is below the user-specified threshold, max-dist. The final transmission clusters can be further analyzed with phylogenetic software.

To quickly identify putative outbreaks and transmission clusters, we developed BREAKFAST, a tool for rapid sequence clustering in the specific context of SARS-CoV-2, and applied it to German and international sequences. Our approach, which derives transmission clusters from SNP occurrences, is motivated by the low mutation rate of SARS-CoV-2 [16]. Here, the pairwise genetic distance between multiple sequences is computed via a constructed sparse matrix of alignment-based genomic profiles. Clusters are defined by identifying chains of sequences whose pairwise distance is below a user-defined threshold (see Figure 2).

Using pre-computed mutation profiles, we clustered 120,000 sequences in 65 s using 100 cores and a peak of 1.3 GB of RAM. Its efficiency and intuitive parameters make BREAKFAST suitable for monitoring fast-growing clusters and analyzing potential outbreaks on a daily basis. Subsequently, computationally intensive phylogenetic tools can be applied to a smaller set of sequences of interest based on the clustering results.

We demonstrate that targeted methods, which leverage a pathogen’s specific properties, can be used in conjunction with large datasets to provide key insights into the ongoing COVID-19 pandemic. Our approach was applied to add individuals to already known outbreaks and triggered follow-up epidemiological investigations of transmission clusters.

BREAKFAST is freely available at https://github.com/rki-mf1/breakfast (accessed on 1 May 2022).

### 2.2. Viral Emergence and Surveillance

This session was chaired by EVBC member Magda Bletsa (National and Kapodistrian University of Athens, Greece) Two keynote speakers were invited on this topic: Emma Hodcroft (University of Bern, Switzerland) opened the session with a talk on phylogenetics, pandemics, and what comes next (see Section 2.2.1); followed by a talk by Daniel Streicker (University of Glasgow, UK), about whether genomics can help prevent viral emergence. From the submitted abstracts, we selected a talk by John Juma (University of Western Cape, Kenya) on the genomic surveillance of Rift Valley fever (see Section 2.2.2).

#### 2.2.1. Real-Time to Real-Life: Phylogenetics, Pandemics, and What Comes Next (by Emma Hodcroft)

Since the announcement of the first variant of concern (VoC) in December 2020, the COVID-19 pandemic has been increasingly shaped not only by viral spread, restrictions, and immunity, but also by variants with increased transmission and immune evasion. Detecting and tracking these emerging variants—and deciding how to react to them—has been no small challenge. With over 7 million publicly available sequences, and millions of unique clusters of sequences, identifying those with mutations of interest and determining if they might be the next VoC is far from straightforward. As the pandemic progresses, heterogeneity in immune history, through infections, vaccinations, and boosters, also means increasing heterogeneity in how ‘concerning’ a VoC may be: the impact of Omicron varied widely across countries. In turn, future variants on the ‘road to endemicity’ may pose different risks to different populations.

Though it is impossible to predict what future variants may mean for how much SARS-CoV-2 continues to impact society, the return of pathogens that were suppressed during the restrictions of 2020 and early 2021 are a reminder of the common disparity in data and understanding between SARS-CoV-2 and the world of viruses we live in. How do we pivot our real-time test of the role that sequencing, modeling, and immunity panels can play in public health to a sustainable real-life integration of research and healthcare for a better understanding of human viruses overall?

#### 2.2.2. Genomic Surveillance of the Rift Valley Fever: From Sequencing to Lineage Assignment (by John Juma)

*John Juma, Vagner Fonseca, Samson Limbaso, Peter van Heusden, Kristina Roesel, Rosemary Sang, Bernard Bett, Alan Christoffels, Tulio de Oliveira, and Samuel Oyola contributed to this work.*

**Figure 3:**
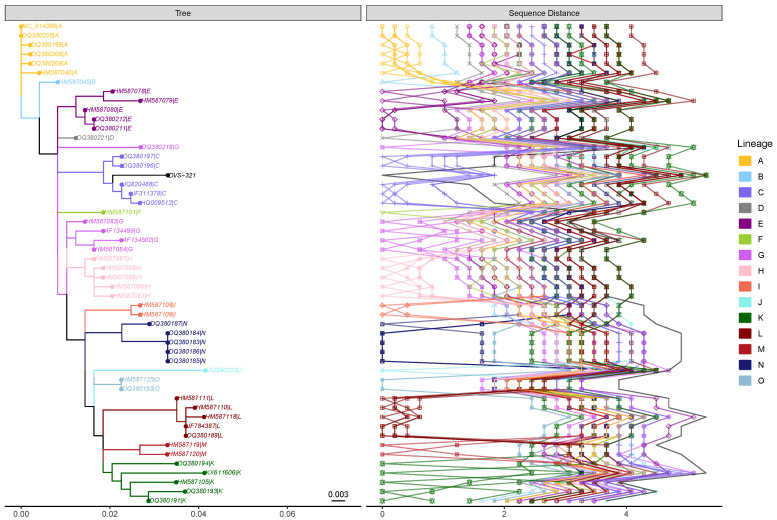
Maximum likelihood phylogenetic tree. Maximum likelihood phylogenetic tree indicating the different clades corresponding to the fifteen major lineages and showing where the query sequence (DVS-321) clusters in the tree. Pairwise distance measure for the different lineages and query samples indicate a genetic diversity, which indicates a maximum diversity of 5% at the nucleotide level.

Genetic evolution of the Rift Valley fever virus (RVFV) in Africa has been shaped mainly by the environmental changes such as abnormal rainfall patterns, climate change, and land subsidence that occurred over the last few decades. These gradual environmental changes are believed to have effected gene migration from macro (geographical) to micro (reassortment) levels. Presently, 15 lineages of RVFV have been identified to be circulating within the sub-Saharan Africa (see Figure 3). International trade in livestock and movement of mosquitoes are thought to be responsible for outbreaks outside endemic regions. Virus spillover events contribute to outbreaks, as was demonstrated by the largest epidemic of 1977 in Egypt. On numerous occasions, viruses from these lineages have been detected outside enzootic regions through probable movement of infected animals and/or mosquitoes. This has led to large outbreaks in countries where the disease had not been previously reported. Genomic surveillance of the virus diversity is crucial in developing intervention strategies. Therefore, we have developed a user-friendly computational tool for rapidly classifying and assigning lineages of partial or whole genome sequences of the virus using the glycoprotein Gn/G2 gene within the M-segment. The computational method is presented both as a command line tool and a web application hosted at https://www.genomedetective.com/app/typingtool/rvfv/ (accessed on 1 May 2022). A user can provide up to 4000 multi-FASTA sequences. Validation of the tool has been performed on a large dataset comprising of partial and whole genome sequences obtained from public database. The Rift Valley Virus typing tool was able to correctly classify all 129 RVFV sequences at species level with 100% specificity, sensitivity and accuracy. All the sequences in lineages A (n=13), B (n=1), C (n=44), D (n=1), E (n=7), F (n=1), I (n=2), J (n=1), M (n=2), N (n=13) and O (n=2) were correctly classified at phylogenetic level, with accuracy, sensitivity, and specificity of 100%. We further validated our tool using genomic data we obtained through sequencing following RVF outbreaks. The tool is useful in tracing the origin of outbreaks and supporting surveillance efforts.

### 2.3. Virus–Host Interactions

This session was chaired by EVBC member Kevin Lamkiewicz (Friedrich Schiller University, Germany). Friedemann Weber (Justus-Liebig University Gießen, Germany) was invited for a keynote talk to speak about diverse anti-interferon strategies by members of the genus phlebovirus (see Section 2.3.1). From the submitted abstracts, we selected talks by Andreas Gruber (University of Konstanz, Germany) about a toolbox for studying RNA virus–host factor interactions (see Section 2.3.2) and by Christopher Jürges (University of Würzburg, Germany) on multi-omics revealing principles of gene regulation and pervasive non-productive transcription in the human cytomegalovirus genome.

#### 2.3.1. Diverse Anti-Interferon Strategies by Members of the Genus Phlebovirus (by Friedemann Weber)

The genus Phlebovirus (order Bunyavirales, tri-segmented negative strand RNA genome) contains species covering a wide spectrum of virulence. Rift Valley fever virus (RVFV), for example, is highly pathogenic, whereas the Sandfly fever Sicilian virus (SFSV) displays an intermediate level of virulence. Although the importance of the mosquito-borne phleboviruses is increasingly recognized, we are only beginning to understand their mechanisms of pathogenicity. A key virulence factor of phleboviruses is the non-structural protein NSs, an inhibitor of the antiviral type I interferon (IFN) system [18]. Our group has identified the mechanisms by which the NSs proteins of both RVFV and SFSV (i) inhibit the transactivation of the IFN genes and (ii) abrogate the antiviral protein kinase R (PKR) pathway. For RVFV, the NSs was found to recruit several E3 ubiquitin ligases of the F-Box type in order to destroy the general host cell transcription factor TF-IIH [19] as well as PKR [20], an antiviral mRNA translation inhibitor. For SFSV, by contrast, the NSs is occluding the DNA-binding domain of the IFN transcription factor IRF-3 to inhibit IFN induction [21,22], and NSs also binds and reprograms the translation initiation factor eIF2B to immunize the ribosomal machinery against PKR signaling [23,24].

Thus, our investigations have demonstrated two surprisingly different IFN escape strategies by these related phleboviruses. While the highly virulent RVFV destroys key host factors of innate immunity, the more benign SFSV only sequesters them.

#### 2.3.2. Staying below the Radar and Exploiting the Host—A Toolbox for Studying RNA Virus—Host Factor Interactions (by Andreas J. Gruber)

**Figure 4:**
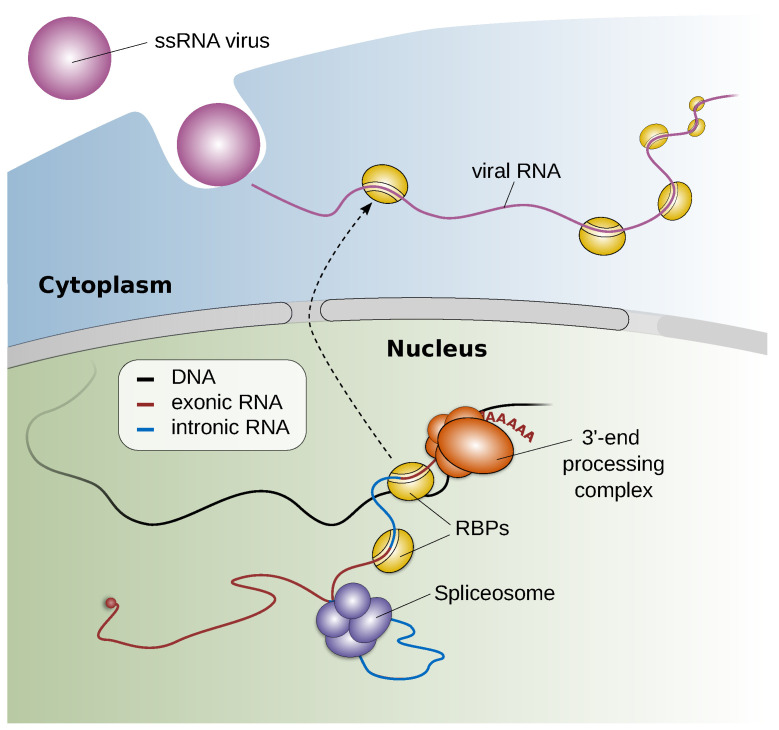
Virus–host factor interactions impact the gene expression of the host cell. Single-stranded RNA (ssRNA) viruses enter the cell and release their RNA genomes into the cytoplasm of the host cell. Viral RNAs can contain binding sequences for host RNA binding proteins (RBPs). The binding of host RBPs to the viral RNA can have proviral or antiviral effects. The sequestration of RBPs by cytoplasmic viral RNA was reported to cause changes in host cell RNA splicing, polyadenylation, and stability [25].

Because viruses require their host cell to reproduce, they have evolved various mechanisms to interact with host factors, such as RNA binding proteins (RBPs). Previous studies have demonstrated that virus–host RBP interactions can have pro- or antiviral effects. Moreover, the sequestration of host RBPs by viral RNA was reported to cause changes in host cell pre-mRNA splicing and polyadenylation as well as mRNA stability, which suggests that virus–host factor interactions can impact the gene expression of the host cell in various ways [25] (see Figure 4). However, the incidence of such virus–host interactions and the host RBP interactomes are, for many viruses, largely unknown. To facilitate the study of RNA virus–host factor interactions, we have developed SMEAGOL, which enables us to identify RBP binding motifs that are enriched or depleted in RNA viral genome sequences. SMEAGOL is available via GitHub (https://github.com/gruber-sciencelab/SMEAGOL (accessed on 1 May 2022)). In order to provide the community with a comprehensive overview of potential single-stranded RNA (ssRNA) virus-RBP interactions, we have applied SMEAGOL to 197 ssRNA virus genomes [26].

To infer RBP binding motifs that can explain global changes in cellular splicing and polyadenylation, we have developed a computational approach called MAPP, standing for Motif Activity on Pre-mRNA Processing [27]. Besides many other applications, in future, the MAPP will support studies that aim to identify RBPs that cause changes in cellular splicing and/or polyadenylation due to their sequestration by viral RNAs. Moreover, as SMEAGOL, MAPP is available via GitHub (https://github.com/gruber-sciencelab/MAPP (accessed on 1 May 2022)). Our tools and analyses provide insights into the RNA virus–host RBP interaction landscape and aim to support future studies that explore virus–host interactions and their potential impact on host RNA splicing and polyadenylation, ultimately feeding into the development of better treatments.

### 2.4. Viral Sequence Analysis

This session was chaired by EVBC member Daniel Todt (Ruhr University Bochum, Germany). Esteban Domingo (Centro de Biología Molecular “Severo Ochoa”, Spain) was invited for a keynote talk and gave a retrospective on the origins and implications of the quasispecies concept (see Section 2.4.1). From the submitted abstracts, we selected talks by Muriel Ritsch (Friedrich Schiller University Jena, Germany), presenting a guidance to store virus sequence and knowledge (see Section 2.4.2); Spyros Lytras (MRC—University of Glasgow Centre for Virus Research, UK), speaking about the dinucleotide composition of the Flaviviridae explored with DinuQ [28]; and Alexander Henoch (Friedrich Schiller University Jena, Germany), speaking about genotype-based classification of IAV to unravel reassortment candidates.

Spyros Lytras was selected for the Best ECR Talk Award. Gabriel Lencioni Lovate (Friedrich Schiller University Jena, Germany) was selected for Best Poster Award, presenting reproducible RNA–RNA interaction probing for RNA proximity ligation data with RNAswarm (see Section 2.4.3).

#### 2.4.1. Origins and Implications of the Quasispecies Concept (by Esteban Domingo)

*Esteban Domingo, Carlos García-Crespo, and Celia Perales contributed to this work.*

Viral quasispecies refers to the complex and dynamic collections of mutants present in individual samples of RNA (and many DNA) viruses [29]. Mutant input is fueled by error rates during template copying that are nearly one million-fold larger than those exhibited by the replicative DNA polymerases of their host organisms. Discovered in the pre-nucleotide sequencing times, the extent of the complexity of mutant swarms in viral populations has been fully appreciated with the application of deep sequencing methodologies. Mutant spectra are generated within individual infected cells, and then they become the substrate for further evolutionary events within each individual host, and then in successive individuals during outbreaks and epidemic expansion. Mutant ensembles may behave as units of selection, and virus adaptation is presently viewed as the replacement of mutant subpopulations by others that are better fit to respond to an environmental change. Positive and negative selection are integrated with random drift prompted by bottleneck events within infected cells, organisms, and during viral transmission. Quasispecies dynamics can be regarded as a paradigm of the pervasive diversity and complexity of the biosphere increasingly evidenced by meta-genome and single cell analyses.

**Figure 5:**
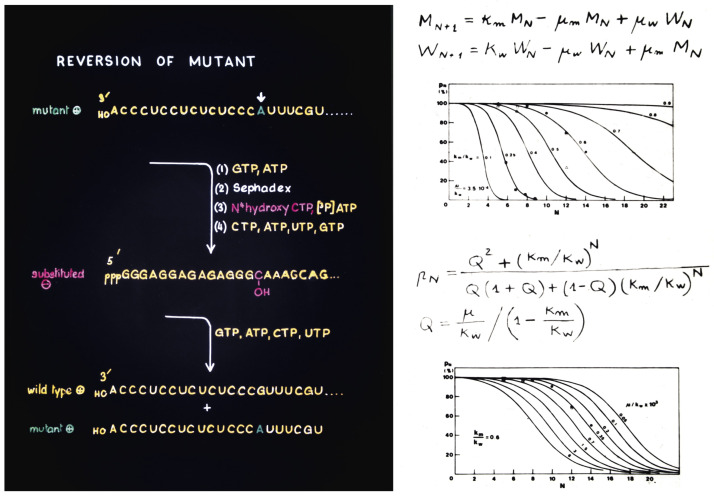
Origins and implications of the quasispecies concept. Images of the work in Charles Weissmann’s laboratory in Zürich in the 1970s. (**Left**): a slide drawn by Weissmann outlining a reversion experiment by site-directed mutagenesis [33]; it is interesting that the N4-hydroxy-CTP used as mutagenic nucleotide is the active component of molnupiravir, presently used as lethal mutagen for SARS-CoV-2. (**Right**): a page of the notebook of Domingo with the experimental data and mathematical predictions of competition between the wild type Qβ phage and the infectious extracistronic mutant (top of page), and reversion of the mutant upon multiplication in *E. coli* (bottom of page) [31,32]; explained also in reference [35].

Quasispecies had two independent origins. One was the development of quasispecies theory by Manfred Eigen and Peter Schuster in Göttingen in the 1970s as a model for the origin of life [30]. The second was the experimental calculation of the mutation rate of bacteriophage Qβ and evidence that its populations consisted in mutant clouds, that took place in the laboratory of Charles Weissmann in Zürich at about the same time [31,32]. The results were possible thanks to a pioneer method of site-directed mutagenesis [33] that produced a viable Qβ mutant [34], whose reversion rate was calculated [31], in a very early precedent of the fitness assays that we now perform routinely in experimental evolution (images of that time reproduced in Figure 5). The historical developments and the implications of quasispecies dynamics for our understanding of RNA viruses and disease mechanisms have been recently reviewed [35].

#### 2.4.2. A Guidance to Store Your Virus Sequence and Knowledge (by Muriel Ritsch)

Currently, virus genome sequences are stored either in NCBI or specific databases, such as ViPR, the HIV database, or GISAID [36,37]. These databases contain a fraction of errors, which can appear before submission (sample contamination or assembly mistakes), during submission (misclassification), or even years after submission (taxonomy adjustment). NCBI and many other general databases do not reliably check whether all uploaded data are correct. Most new entries in these databases are compared by sequence similarity to existing ones, and the mistakes in the databases can cascade. Large-scale, downstream, and evolutionary analysis are hardly possible. Even with much effort and time, filtering true from false entries is not always possible. Good scientific research using these public virus genome databases is further complicated when the metadata or sequences are only partially correct, especially if one extrapolates the growth of viral data [38,39]. To prevent the problem of false-positive sequences in the databases, we propose a guideline for uploading sequences.

Out of this knowledge, here are four main and several side steps that should be followed during uploading sequences: (1) it seems trivial, but appears as a major source of mistakes: naming your virus sequence (existence of the virus, spelling, following the ICTV, and connecting to renamings in the past), (2) assignment of the correct taxa (ICTV as ground truth), (3) supply of necessary metadata, and (4) control sequence. The last step, especially, is essential because there are many viral sequences with non-viral dangling ends. Database entries that do not follow these steps can lead to incorrect conclusions and even jeopardize entire studies.

For future sequence uploads, alignments and quality checks should be conducted (ideally performed by the database) to predict whether the entire sequence is correct. Such alignments should be built with other known viruses of the same taxon. Additionally, the problem of legal issues related to virus databases should be tackled. We envision a future database containing an easy-to-use interface, quality check, a private workspace, and tools for assembly, alignment, and phylogeny analysis with SOPs in the field.

#### 2.4.3. Reproducible RNA–RNA Interaction Probing for RNA Proximity Ligation Data with RNAswarm (by Gabriel Lencioni Lovate)

*Gabriel Lencioni Lovate, Celia Jakob, Hardin Bolte, Kevin Lamkiewicz, Martin Schwemmle, and Manja Marz contributed to this work. Gabriel Lencioni Lovate was selected for Best Poster Award.*

Influenza A viruses (IAVs) have a segmented RNA genome that has to be correctly packaged to produce infective viral particles. Each of IAV’s genome segments are organized as discrete RNA–protein complexes, called viral ribonucleoproteins (vRNP). The genome packaging process is selective and depends on interactions between individual vRNPs, potentially mediated by their RNA portion. These RNA–RNA interactions can be probed on a large scale through RNA proximity ligation methods. The approach consists of linking interacting RNA molecules via chemical cross-linking, followed by high-throughput sequencing (HTS) of the interacting RNAs. The HTS reads have to be then split-mapped to the viral genome to identify interacting regions.

To improve the understanding of the RNA–RNA interactions that might play a role in IAV’s genome packaging, we present RNAswarm, a novel bioinformatics pipeline that is expanding the scope of high-throughput analyzes of RNA–RNA interactions. In particular, RNAswarm allows for statistically comparing the frequency of RNA interactions among different strains or experimental settings. Thus, RNAswarm offers virologists an automated and reproductive method for prioritizing and comparing the RNA–RNA interactions, a time-consuming job prone to individual biases when performed manually.

### 2.5. Virus Identification and Annotation

This session was chaired by EVBC member Alba Pérez-Cataluña (Instituto de Agroquímica y Tecnología de Alimentos, Spain), one of the ViBioM 2022 organizers. From the submitted abstracts, we selected talks by Jiarong Guo (Ohio State University, United States), presenting a multi-classifier, expert-guided approach to detect diverse DNA and RNA viruses (see Section 2.5.1); and Enrique González-Tortuero (University of Salford, UK), speaking about the evaluation of gene-calling programs for viral genome annotation (see Section 2.5.2).

#### 2.5.1. VirSorter2: A Multi-Classifier, Expert-Guided Approach to Detect Diverse DNA and RNA Viruses (by Jiarong Guo)

**Figure 6:**
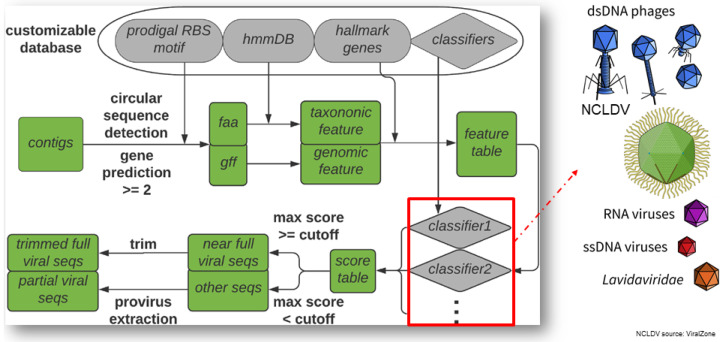
Overview of the viral sequence prediction pipeline used in VirSorter2 [40]. The “hmmDB” includes viral protein HMMs from two of the largest databases, VPF and Efam [38,42]. Distinct classifiers (random forest) are built for each of five major viral groups to improve accuracy on diverse viruses. Adapted with permission from [40] (https://creativecommons.org/licenses/by/4.0/). Copyright 2021, Guo et al.

Viruses have been demonstrated to play an important role in many biospheres, ranging from ocean, soil, to human ecosystems with the advent of meta-omics; however, identifying viral sequences from large sequencing data mixed with host sequences is still a challenging task, as viruses do not have universal marker genes and also lack representatives in existing reference databases. Most existing tools also lack the capability to detect viruses other than bacteriophage. Here, we introduce VirSorter2 [40] (see Figure 6), with major updates to the original VirSorter [41] including (1) integrating machine learning techniques and expanding the predicting features from 6 in the original VirSorter to 27; (2) dividing the global viral sequence spaces into five major groups (dsDNA phage, ssDNA, RNA virus, giant virus [NCLDV, Nucleocytoviricota], and lavidaviridae) and building a distinct classifier for each group; (3) leveraging large viral protein hidden markov model (HMM) profile databases from diverse ecosystems [38,42] and also expert curated high quality viral genomes sequences collected from isolates and environmental metagenomes to improve the ability to detect diverse and novel viruses; (4) incorporating modern workflow management tool (snakemake) [43] for improved scalability in high performance computing clusters and also overall usability. In the benchmark with genomes from both isolated and uncultivated (from metagenome) viruses, VirSorter2 uniquely demonstrated consistent high accuracy (F1-score > 0.8) in all five viral groups, while other tools performed poorly with viral groups other than dsDNA phage, which is best represented in the reference databases. VirSorter2 can also uniquely minimize false detection of eukaryotic and plasmid sequences as viral. Further, VirSorter2 has a modular design and provides functions to add more classifiers to keep high accuracy, as we discover more diversity of viral sequence space. In conclusion, VirSorter2 demonstrates that its multi-classifier and modular design can enable high performance to detect diverse viruses, and will be a useful tool to advance our knowledge of virus ecology and evolution. To best serve the research community, we maintain a “live protocol” (https://dx.doi.org/10.17504/protocols.io.bwm5pc86 (accessed on 1 May 2022)) for best practices on using VirSorter2 for virus sequence identification, including curating less well-studied viruses and mobile genetic elements, and establishing bona fide virus-encoded auxiliary metabolic genes.

#### 2.5.2. Evaluation of Gene-Calling Programs for Viral Genome Annotation (by Enrique González-Tortuero)

Due to the development of the next-generation sequencing platforms and genome analysis tools, newly available viral genomes and metagenomes have increased exponentially. Genome annotation pipelines rely primarily on gene-calling software, which identifies genes regardless of the sequence taxonomic background. Although gene-calling programs provide a rapid genome annotation, they can misidentify genes and start codons, propagating and perpetuating errors over time. This study assessed the performance of multiple gene-calling programs for viral genome annotation against the entire RefSeq viral database. MetaProdigal [44] and FragGeneScan [45] were the most accurate programs for DNA and RNA viruses (101.01% and 99.62%, respectively) according to the number of coding genes. By considering the coordinates of the coding genes, Prodigal [46] scored high for DNA viruses (83.92%), while GeneMarkS [47] generated the most reliable results for RNA viruses (60.84%). The quality of the coordinates predicted for RNA viruses was poorer than for DNA viruses, suggesting the need to develop gene-calling programs to deal with RNA viruses. Additionally, none of the gene-calling programs reached 90% accuracy for gene prediction of DNA viruses. The use of Prokka [48] for the genome annotation of giant viruses, bacteriophages, and viruses of Archaea might explain the highest score of Prodigal when predicting genes in DNA viruses. Manual curation should improve any automatic annotation, especially by validating the presence of these genes with wet-lab experiments. This evaluation of the current gene-calling programs might help improve viral genome annotation pipelines and highlight the need for more expression data to improve the rigour of reference genomes.

### 2.6. Phages

This session was chaired by EVBC member Noriko Cassman (Friedrich Schiller University, Germany). Evelien Adriaenssens (Quadram Institute Bioscience, UK) was invited as keynote speaker to talk about phages in the human gut: a taxonomist’s perspective. From the submitted abstracts, we selected talks by Dimitri Boeckaerts (Ghent University, Belgium) about dual identification of novel phage receptor-binding proteins based on protein domains and machine learning (see Section 2.6.1); and Antoni Luque (San Diego State University, United States) about the prediction of viral capsid architectures from metagenomes (see Section 2.6.2). Célia Pas (Ghent University, Germany) was selected for Best Poster Award presenting a blueprint of tail fiber modularity and its relationship with host specificity for STEC serovars (see Section 2.6.3).

#### 2.6.1. Dual Identification of Novel Phage Receptor-Binding Proteins Based on Protein Domains and Machine Learning (by Dimitri Boeckaerts)

*Dimitri Boeckaerts, Michiel Stock, Bernard De Baets, and Yves Briers contributed to this work.*

**Figure 7:**
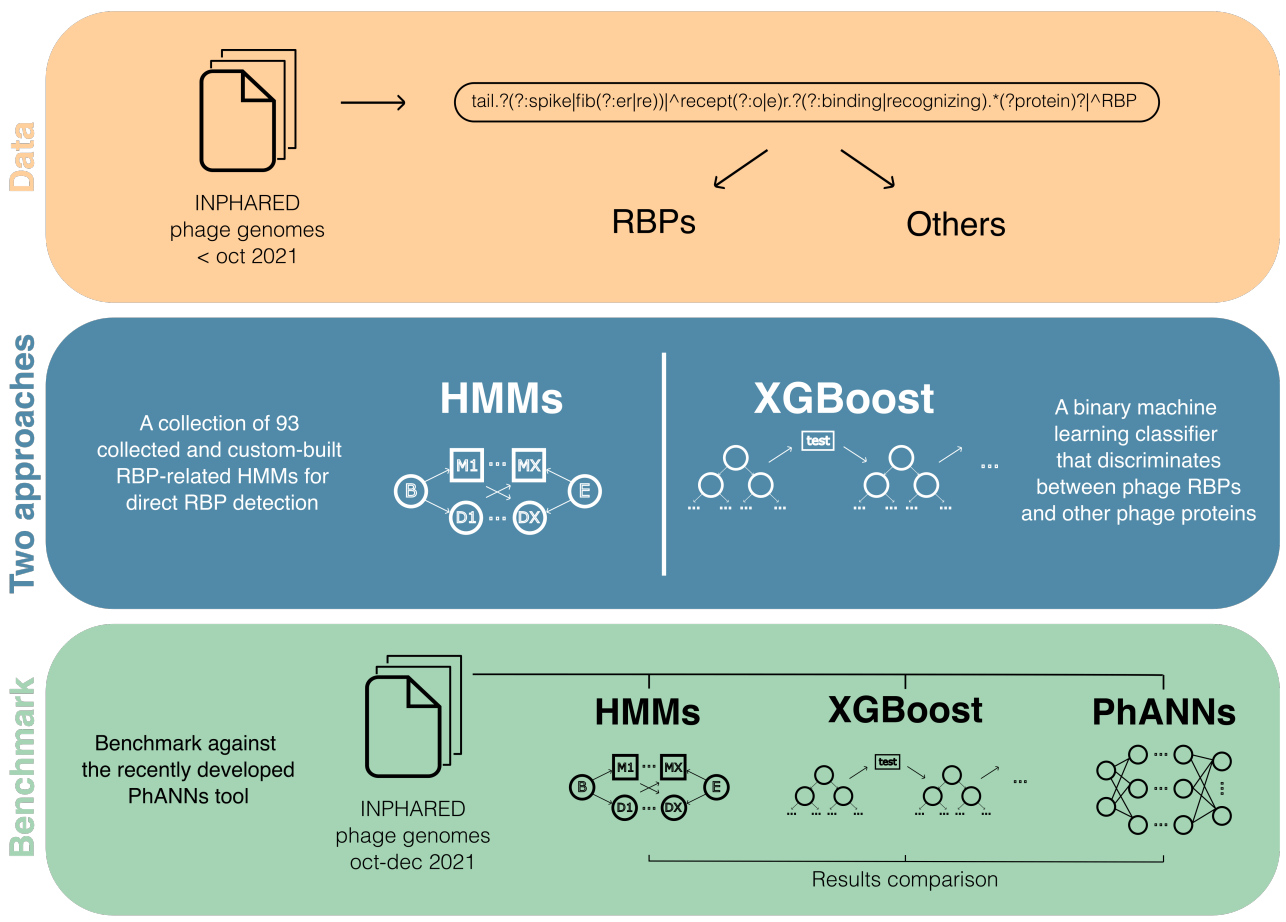
Dual identification of novel phage receptor-binding proteins. Graphical abstract of the collected phage genome data, the developed RBP detection tools and the benchmark against the recently developed PhANNs tool [50].

Bacteriophages (phages for short) are an emerging alternative treatment against multi-drug resistant bacteria. Their often-narrow host specificity is an additional benefit with regards to side-effects on healthy microbiota, but often necessitates a labor- and time-intensive search of phages that match a specific pathogen. To circumvent this problem, synthetic biology methods can be applied to precisely engineer the specificity of phages towards their bacterial hosts. For example, receptor-binding proteins (RBPs) can be modified or swapped between phages to adjust or broaden the narrow host specificity [49]. Today, the amount of publicly available phage genome data is steadily increasing, presenting opportunities to study phages in new ways, including their RBPs. However, many different annotations exist for RBPs, and many phage proteins are not even annotated at all. PhANNs, a recently developed machine-learning-based tool, has started to bridge this gap by predicting ten major classes of phage proteins [50]. From their research, tail fiber proteins (a subset of RBPs) appear among the most difficult classes to predict. To further address this lacking or inconsistent annotation, we have developed two parallel approaches specifically for the complex identification of RBP sequences in publicly available phage genome data (see Figure 7). Our first approach consists of a collection of RBP-related hidden Markov models (HMMs) that were both collected from the Pfam database as well as custom-developed [51]. These HMMs represent RBP-related conserved protein domains that can be used to detect RBPs. Secondly, we have trained an Extreme Gradient Boosting model that classifies sequences into two categories: phage RBPs and other phage proteins [52]. Both methods start from a comprehensive data processing that identifies the different annotation keywords associated with RBPs. We show that both approaches can be complementary to one another and can be used together to identify RBP sequences in genomic data. Finally, we have benchmarked our methods against PhANNs. Our best-performing model reached a precision-recall area-under-the-curve of 93.8% and outperforms PhANNs on an independent test set, reaching an F1-score of 84.0% compared to 69.8%. We aim to publish this work and open source the code and database for the research community to freely use and build upon.

#### 2.6.2. Predicting Viral Capsid Architectures from Metagenomes (by Antoni Luque)

*Antoni Luque, Diana Y. Lee, Sean Benler, Colin Brown, Caitlin Bartels, Katelyn McNair, Stephen Nayfach, Simon Roux, Manal A. Swairjo, Robert A. Edwards, and Simon White have contributed to this work.*

**Figure 8:**
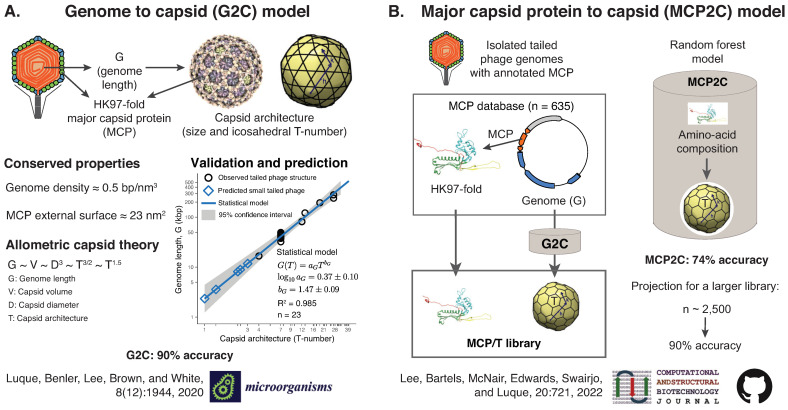
Models predicting capsid architecture. (**A**) The genome to capsid (G2C) model relies on the conserved properties of tailed bacteriophages. The model uses the genome length to predict the capsid architecture (diameter and icosahedral T-number). (**B**) The major capsid protein to capsid (MCP2C) relies on the G2C model to build a library of putative capsid architectures and MCPs from isolated tailed phage genomes. It predicts the capsid architecture of tailed phages directly from the major capsid protein sequence. The current G2C and MCP2C python versions are accessible at https://github.com/luquelab/Lee_etal_CSBJ_2022/tree/main/3_executables (accessed on 1 May 2022).

Viruses protect their genome in protein shells called capsids assembled from multiple copies of the same protein. However, it is unclear what molecular protein adaptations have promoted the stability of viral capsids across environments. $10^{31}$ viral particles are evolving on the planet at a given time. This number dwarfs the few thousand cultured and uncultured viruses observed under the microscope [53,54] and the few hundred high-resolution capsids reconstructed molecularly [55]. To tackle the challenge of investigating the adaptation of capsids to different environments, the Luque lab is developing biophysical-based models to predict the physical architecture of viral capsids directly from cultured and uncultured viral sequence data (see Figure 8). The approach builds on the conserved genomic and architectural properties of viruses that assemble their capsid from proteins sharing the same capsid protein fold [56,57].

Our initial models focused on tailed phages, which infect bacteria and are the most abundant viruses. Tailed phages assemble their icosahedral capsids from proteins adopting the HK97-fold and pack their double-stranded DNA genome at high densities [58,59]. These conserved properties predict an allometric law for the viral genome length and the capsid architecture (size and T-number) [60]. The analysis of 23 high-resolution capsid structures confirmed the theoretical relationship and led to the genome to capsid (G2C) model with 90% accuracy (see Figure 8A). The model was applied to 3348 isolated tailed phage genomes from NCBI RefSeq, and 1496 metagenomically assembled (putatively) complete genomes from the human gut [60,61]. The G2C model identified tailed phage candidates adopting small capsids (T ≤ 3) that have not been previously reconstructed but may hold the key to elucidating the origin of tailed phage capsids.

The G2C model relies on the genome length to make predictions. However, most assembled viral genomes in metagenomes are incomplete [62]. To circumvent this issue, we developed a model predicting the capsid architecture directly from the protein sequence of the major capsid protein, the MCP2C model (see Figure 8B). The application of the G2C model to 635 isolated phage genomes–containing a validated HK97-fold MCP–built a sufficiently large library of MCP sequences and capsid architectures for statistical training [63]. The MCP’s amino-acid frequency was used to train the statistical learning method random forest. MCP2C yielded a 74% accuracy in predicting the capsid architecture, and our projections indicate that a library with 2500 entries would deliver a 90% accuracy. The application of the MCP2C model to 1479 HK97-fold MCPs predicted a significantly large amount (~15%) of jumbo phage capsid architectures (T ≥ 25) in human gut metagenomes [63].

Both models (G2C and MCP2C) could be used for other HK97-fold-based viruses that pack the genome at high densities, such as Herpesvirales [56]. However, predicting capsid architectures for non-HK97-fold based viruses would require applying the analogous approach described above for each capsid protein fold’s viral lineage [64].

#### 2.6.3. A Blueprint of Tail Fiber Modularity and Its Relationship with Host Specificity for STEC Serovars (by Célia Pas)

*Célia Pas, Lars Fieseler, and Yves Briers contributed to this work. Célia Pas was selected for Best Poster Award.*

Shiga toxin-producing *E. coli* (STEC) is a severe foodborne pathogen belonging to the critical priority pathogen list defined by the World Health Organization. The use of broad-spectrum antibiotics does not only contribute to the spread of antibiotic resistance, but in the case of this specific pathogen also induces the release of the Shiga toxin. Phages, as the natural predator of bacteria, therefore offer great potential in STEC treatment. The phage–host relationship is very specific and complex, where tail fibers or tailspikes of the phages are the first phage proteins initiating the infection process. These proteins bind to various outer membrane structures including O-antigen, a serovar specific component on the lipopolysaccharide layer of the bacterial cell wall. Tail fibers with O-antigen binding properties were identified in multiple phage species such as *Kutter*-, *Uetake*-, *Lederberg*-, *Gamaleya*-, and *Kagunaviruses*. Moreover, we confirmed that tail fibers are occasionally passed on to entirely different phage families by horizontal gene transfer, allowing the phages to infect specific STEC serovars. This method of screening for new O-antigen-specific tail fibers is highly interesting to develop serotype-targeting microbials, especially in current times, where antimicrobial resistance is a serious threat to global health and development.

### 2.7. Viral Diversity

This session was chaired by EVBC director and ViBioM 2022 organizer Manja Marz (Friedrich Schiller University, Germany). Matthew Sullivan (Ohio State University, United States) was invited as a keynote speaker to talk about ocean viruses: patterns, processes, and paradigms on a planetary scale (see Section 2.7.1). From the submitted abstracts, we selected talks by Daan Jansen (KU Leuven, Belgium) about community-typing as a way to explore virome compositional changes in IBD patients (see Section 2.7.2); and Alex Veglia (Rice University, United States) about an automated virus amplicon sequence analysis program to support investigations of viral community ecology. Luca Nishimura (SOKENDAI, Japan) was selected for Best Poster Award, presenting a virome analyses of the ancient individuals who lived in the Japanese archipelago 3000 years ago (see Section 2.7.3).

#### 2.7.1. Ocean Viruses: Patterns, Processes, and Paradigms on a Planetary Scale (by Matthew Sullivan)

Microbes are recently recognized as driving the energy and nutrient transformations that fuel Earth’s ecosystems in soils, oceans, and humans. Where studied, viruses appear to modulate these microbial impacts in ways ranging from mortality and nutrient recycling to extensive metabolic reprogramming during infection. As environmental virology strives to get a handle on the global virosphere (the diversity of viruses in nature), we face challenges to organize this ‘sequence space’ (create a sequence-based viral taxonomy), link these viruses to their natural hosts (who infects whom), and establish how virus populations are structured (ecological drivers) and impact natural ecosystems (their impacts). Here, I will share current thinking on how to study viruses in complex communities and how these efforts are revealing new biology, with a particular focus on the patterns, processes, and paradigms emergent from studying the Tara Oceans global datasets. These advances in viral ecogenomics provide fundamental information critical for bringing viruses into ecosystem models, and the new capabilities are empowering a new generation of eco-systems biologists.

#### 2.7.2. Community-Typing as a Way to Explore Virome Compositional Changes in IBD Patients (by Daan Jansen)

*Daan Jansen, Gwen Falony, Sara Vieira-Silva, Kathleen Machiels, Clara Caenepeel, Séverine Vermeire, and Jelle Matthijnssens contributed to this work.*

**Figure 9:**
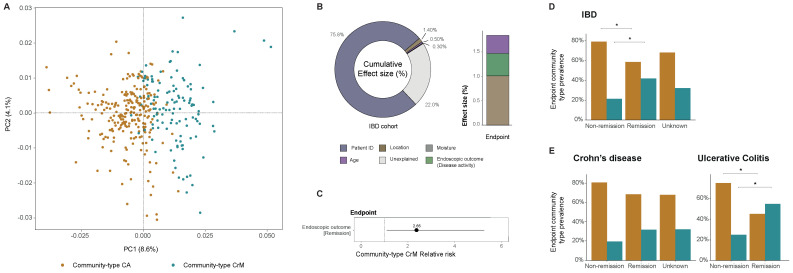
(**A**) Principal coordinates analysis of inter-individual differences of the gut virome (genus level Bray-Curtis dissimilarity) in the IBD cohort (circles colored by viral community-type, n=366). (**B**) Metadata variables significantly correlating to virome compositional variation in both the IBD cohort (left) and post-intervention samples (right) (dbRDA, genus-level Bray-Curtis dissimilarity), as determined by a multivariate linear regression model. (**C**–**E**) Modeling the association between the metadata drivers of post-intervention samples and the prevalence of viral community-type CrM (logistic regression, n=166, only significant associations shown). (**C**) Relative risk ratio of prevalence of viral community-type CrM with the significant driver (endoscopic remission) of virome variation. (**D**) Representation of viral community-type prevalence in post-intervention samples (n=166) stratified according to the endoscopic outcome (non-remission, remission, unknown). (**E**) Representation of viral community-type prevalence in post-intervention samples per IBD subtype, CD (left, n=115) and UC (right, n=51) stratified according to the endoscopic outcome (non-remission, remission, or unknown). * p<0.05 (adjustment for multiple testing was performed using the Benjamini-Hochberg methods).

Inflammatory bowel diseases (IBD) are a group of chronic inflammatory diseases of the gut. It is commonly divided into two major variants, ulcerative colitis (UC) and Crohn’s disease (CD). The pathophysiology is unknown; however, it is thought to result from an aberrant immune reaction to the commensal gut microbiota. Community-typing is a common practice in bacteriome analysis allowing for the stratification of individuals based on their gut microbiome (e.g., ‘enterotyping’) [65,66]. Similarly, the viral counterpart of these enterotypes might allow the stratification of individuals based on their gut virome. The aim of the present study is to use community-typing as a tool to explore virome compositional changes in IBD patients. Fecal samples were selected from 181 patients undergoing immunomodulatory therapy, and a baseline (pre-intervention) and primary endpoint (post-intervention) sample was collected for each patient. Viral metagenomics and deep sequencing were performed following viral enrichment with the NetoVIR protocol [67]. Briefly, quality-controlled reads were de novo assembled into contigs using MetaSPAdes [68]. Clustering of the contigs was performed to remove redundancy and obtain a set of non-redundant (NR) contig at 95% average nucleotide identity and 80% coverage [62]. Abundances were calculated per sample by mapping quality-controlled reads to the set of NR contigs using bwa-mem2 [69]. Next, bacteriophages identified with VirSorter2 (see Section 2.5.1) and an adequate quality tier (>50%; as determined by CheckV) were selected for further analyses [40,62]. To obtain higher viral taxonomies, phage genomes were clustered into genus-level groups based on pairwise average amino acid identity and gene sharing, yielding 874 genus-level vOTUs [70]. Community-typing of the genus-level (rarefied) abundances with logarithmic transformation was performed based on Dirichlet multinomial mixtures [66]. We were able to condense the gut virota into two community-types, CA and CrM (see Figure 9A, n=363, genus-level, Bray-Curtis dissimilarity). Community-type **CA** demonstrated a low alpha-diversity and a high relative abundance of **Ca**udoviricetes [non-CrAss] phages. Community-type **CrM** demonstrated a high alpha-diversity and a high relative abundance of *Caudoviricetes* [**Cr**Ass] and ***M**algrandaviricetes* phages. Distance-based redundancy analysis (dbRDA) allowed us to determine the metadata affecting the virome composition (Figure 9B, left). The composition was explained by several factors: patients’ individuality (multivariate dbRDA, $R^{2}=75.8$%, p=0.001), disease location (multivariate dbRDA, $R^{2}=1.40$%, p=0.001), age (multivariate dbRDA, $R^{2}=0.50$%, p=0.001), and moisture (multivariate dbRDA, $R_{2}=0.30$%, p=0.007). Interestingly, the virome composition was better explained by disease location than by diagnosis, as shown in previous bacterial research. Moreover, the virome composition was associated to therapeutic response (multivariate dbRDA, $R_{2}=0.46$%, p=0.032) in post-intervention samples (Figure 9B, right). Next, we associated community-types with explanatory metadata (univariate logistic regression, n=166, $R_{2}=3.91$%, Adjp=0.0280) in post-intervention samples and found disease activity (endoscopic remission relative risk = 2.65) to be linked with an increased risk of hosting community-type CrM (Figure 9C). We confirmed that responding IBD patients had a higher percentage of community-type CrM compared to non-responding IBD patients (Figure 9D, n=166, endoscopic remission, 21.2% vs. endoscopic non-remission, 41.7%, $X_{2}=6.30$, Adjp=0.0300). This increase seemed to be majorly driven by UC patients, but not CD patients (Figure 9E, n=51, endoscopic remission UC, 25.0% vs. endoscopic non-remission UC, 54.8%, $X_{2}=4.41$, Adjp=0.0357). These findings suggest that viral community-typing allows for stratification of IBD patients based on their gut virome composition and might be a valuable tool to better understand IBD subtypes or as a potential future biomarker.

#### 2.7.3. Virome Analyses of the Ancient Individuals Who Lived in the Japanese Archipelago 3000 Years Ago (by Luca Nishimura)

*Luca Nishimura was selected for Best Poster Award.*

Ancient DNA has been extracted from historical samples such as bones and teeth. Recently, ancient DNA studies have shed light on ancient people’s genomes and have elucidated the population structures and migration histories at ancient times. Additionally, we can discover the ancient microbial or viral genomic information from the ancient DNA of human remains. Some viruses that existed in ancient people were pathogenic and valuable to understanding the pandemic in ancient times. On the other hand, most were non-pathogenic and related to ancient people’s health. Therefore, it is crucial to analyze those ancient viruses to comprehend viral evolutions and ancient people’s health conditions.

Here, we utilized whole genomic sequencing data of the “Jomon” people, who lived in the Japanese archipelago more than 3000 years ago, to analyze the ancient viral genomes. As a result, we obtained several ancient viral genomic information related to oral commensal bacteria and marine habitats. They might be related to ancient Jomon people’s diet. Moreover, we successfully reconstructed an ancient Siphovirus contig89 phage genome from 3800-year-old specimens and utilized it to construct phylogenetic trees [71]. Our results indicate that the ancient viral genomes are helpful to understand the ancient people’s diet and viral evolution.

## 3. EVBC Annual Meeting

The EVBC was founded in 2017 to bring together experts in virology and virus bioinformatics [1,2] and is constantly growing. Since the last annual meeting in October 2020 [3], 66 new members from 18 different countries joined the EVBC. About 28% of our members are females. After the conference, all speakers were invited to join the EVBC.

EVBC is offering several services to our members and the virus bioinformatics community. We are publishing a monthly newsletter, informing about recent research results, upcoming events, job vacancies and further announcements. Moreover, we are curating a list of specific bioinformatics tools to be applied in virology.

At the last meeting in 2020, we experienced an increase in registrations after announcing the online event. This has made the meeting accessible to a broader range of scientists, in particular younger researchers and researchers newly entering the field. This brought us to the idea to set up a monthly online lecture series ‘viruses in silico’ to keep scientists up to date with the latest developments in virus bioinformatics, especially new tools. This lectures is attended by 20–80 participants each month. In addition, we are organising a monthly ‘Viromics Webinar Series’ for early career researchers studying viruses in complex communities (together with the Center of Microbiome Science at OSU and EMERGE). For the future, we are also planning to set up a workshop program.

In a survey among EVBC members, we were asked to introduce our members. We are now posting two member profiles each month to help you to get to know each other and possibly find interesting collaboration opportunities.

To learn more about our work or to become an EVBC member, please have a look at our website http://evbc.uni-jena.de/ (accessed on 1 May 2022).

## 4. Conclusions

As in previous years, in 2022 members of the community met at the International Virus Bioinformatics Meeting to discuss current research of the field. This report summarizes the presented work and we hope that it will allow the wider community to profit from the meeting by gaining interesting insights into the field of virus bioinformatics and its current state-of-the-art research.

We encourage interested researchers to join us at the next International Virus Bioinformatics Meeting to be held in 2023 in Valencia. For more information, do not hesitate to contact us via evbc@uni-jena.de.
